# PRL-3 is essentially overexpressed in primary colorectal tumours and associates with tumour aggressiveness

**DOI:** 10.1038/sj.bjc.6604747

**Published:** 2008-10-28

**Authors:** D G Molleví, A Aytes, L Padullés, M Martínez-Iniesta, N Baixeras, R Salazar, E Ramos, J Figueras, G Capella, A Villanueva

**Affiliations:** 1 Translational Research Laboratory, Institut Català d’Oncologia-IDIBELL, L’Hospitalet de Llobregat, 08907 Barcelona, Spain; 2 Medical Oncology, Institut Català d’Oncologia-IDIBELL, L’Hospitalet de Llobregat, 08907 Barcelona, Spain; 3 Department of Digestive Surgery, Hospital Universitari de Bellvitge, L’Hospitalet de Llobregat, 08907 Barcelona, Spain; 4 Hepatobilliary surgery unit, Hospital Josep Trueta, Girona 17007, Spain

**Keywords:** PRL-3, phosphatase, stage-III, colorectal carcinoma, metastasis

## Abstract

Phosphatase PRL-3 has been involved in different types of cancer, especially in metastases from colorectal carcinoma (CRC). In this study, we explored both isoforms of PRL-3 as a biomarker to predict the recurrence of stage IIIB-C CRC. Overexpression of PRL-3 was investigated in primary human colorectal tumours (*n*=20) and hepatic metastases (*n*=36) xenografted in nude mice, samples characterised by absence of human non-tumoral cells, showing a high degree of expression in metastases (*P*=0.001). In 27 cases of matched normal colonic mucosa/primary tumour/hepatic metastases, PRL-3 overexpression occurs in primary tumours *vs* normal mucosa (*P*=0.001) and in hepatic metastases *vs* primary tumours (*P*=0.045). Besides, our results in a series of 80 stage IIIB-C CRC primary tumours showed that high levels of PRL-3 were an independent predictor of metastasis (*P*<0.0001; OR: 9.791) in multivariate analysis of a binary logistic regression and that PRL-3 expression tightly correlates with parameters of bad outcome. Moreover, PRL-3 expression associated with poor outcome in univariate (*P*<0.0001) and multivariate Cox models (hazard ratio: 3.322, 95%, confidence interval: 1.405–7.852, *P*=0.006). In conclusion, PRL-3 is a good marker of aggressiveness of locally advanced CRS and a promising predictor of distant metastases. Nevertheless, for prognosis purposes, it is imperative to validate the cutoff value of PRL-3 expression in a larger and consecutive series and adjuvant setting.

Colorectal carcinoma (CRC) is the third most prevalent cancer in the world. When CRC is detected early, the 5-year survival rate is approximately 90%, but only 35–40% of CRC are diagnosed at this stage. In the bulk of the patients, CRC is detected when lymph node metastases are present and a significant number of cases are diagnosed with disease in distant sites, especially in the liver. Despite the clinical importance of metastasis being responsible for most cancer deaths, much remains to be learned about the biology of the metastatic process.

Protein tyrosine phosphatases (PTPs) are key regulatory enzymes in various signal transduction pathways. The PRL phosphatases represent a novel subfamily of PTPs comprising three members (PRL-1, PRL-2 and PRL-3) sharing a high degree (>75%) of amino-acid sequence identity. PRL-1 was originally identified as an immediate early gene in regenerating liver (Mohn *et al*, 1991). Overexpression of PRL-1 and PRL-2 transformed mouse fibroblasts and hamster pancreatic epithelial cells in culture (Cates *et al*, 1996), and promoted tumour growth in nude mice (Diamond *et al*, 1994). *PRL-3* gene, located in 8q24-3, has been involved in processes of migration, invasion and metastasis in different types of cancer (Miskad *et al*, 2004, 2007; Wu *et al*, 2004; Polato *et al*, 2005; Radke *et al*, 2006; Wang *et al*, 2006; Qian *et al*, 2007). Initially, specific overexpression of PRL-3 in colorectal cancer liver metastases (LMs) was observed when compared with matched primary tumours in association with gene amplification (Saha *et al*, 2001; Bardelli *et al*, 2003). Kato *et al*, using *in situ* hybridisation, reported that primary tumours showed variable degrees of PRL-3 expression that associated with lung or liver dissemination (Kato *et al*, 2004). These findings were also reproduced when protein expressed was assessed using non-commercial antibodies. In this study, we aimed to analyse the relative contribution of cancer cells and stroma to the expression of PRL-3 in primary CRC and metastases and to determine whether quantitative RT-PCR (qRT-PCR) could be a good alternative to quantitatively analyse PRL-3 expression in routinely collected specimens and explore their putative clinical usefulness.

## Materials and methods

### Tumour samples

All patients gave written consent to give tumour samples to the Hospital Universitari de Bellvitge tumour bank, and the ethics committee of the hospital cleared the tumour harvesting and grafting protocols.

#### Xenografts

Twenty primary human colorectal tumours and 36 LMs from CRCs were grafted into 5-to-6-week-old male nu/nu Swiss mice weighing 18–22 g (Harlan, Barcelona, Spain). Mice were anaesthetised using isofluorane inhalation. A 2-mm^3^ piece of primary colorectal tumour and LM were anchored to the caecum or the anterior hepatic lobe, respectively. All experiments were approved by the Institutional Animal Care Committee. After a growth period ranging from 2 to6 months, xenografts were excised and immediately frozen in dry ice. These samples are characterised by the absence of non-tumoral human cells because of the mice desmoplastic reaction.

#### Matched microdissected samples

Six sets of matched primary tumour stromal cells, primary tumour cancer cells, LM stromal cells and LM cancer cells corresponding to six different patients were microdissected using the Arcturus-PixCell-II Laser Capture Microdissection System. RNA extraction was performed using the TrizOl reagent and glycogen to increase the yield of the procedure.

#### Matched triplet samples

Twenty-seven matched triplets of fresh-frozen non-adjacent normal colonic mucosa, primary tumour and LM (22 synchronous and five metachronous) were obtained between January 2001 and December 2005 from the Department of Pathology, Hospital Universitari de Bellvitge, Barcelona, Spain.

#### Metastatic CRC (mCRC) and paired non-adjacent normal mucosa fresh-frozen specimens

Eighty primary metastatic colorectal tumours (46 stage IIIB and 34 stage IIIC) and their correspondent normal colonic mucosa were obtained from the Pathology Department of the Hospital Universitari de Bellvitge. Among the 80 CRCs, we classify 43 CRCs (29 stage IIIB and 14 stage IIIC) that did not develop distant metastases for at least 60 months and 37 CRCs (17 stage IIIB and 20 stage IIIC) that developed liver and/or lung metastases.

All patients underwent surgical resection: 70 radical and 10 palliative surgeries. All patients received chemotherapy adjuvant treatment. Median follow-up was 107.30 months (range 43–132). End points were local recurrence for disease-free survival (DFS) and cancer-related death or last follow-up for OS. At the end of the follow-up period, 45 of 80 patients (56.25%) were alive without disease, six (7.5%) were alive with disease and 29 died because of cancer.

### Quantitative real-time RT–PCR analysis

Total RNA was retrieved from all tissue specimens using the TrizOL Reagent method. cDNA was obtained using recombinant ribonuclease inhibitor RNAse OUT and M-MLV retrotranscriptase. Quantitative real-time RT–PCR analyses were performed using the Light-Cycler 2.0 Roche System and the LightCycler FastStart DNA Master SyBR Green I kit (Roche). Oligonucleotide sets for RT–PCR amplification of *PRL-3* were as follows: *PRL-3* isoform 1: up/5′-GGGACTTCTCAGGTCGTGTC-3′; down/5′-AGCCCCGTACTTCTTCAGGT-3′; *PRL-3* isoform 2, up/5′-GGGACTTCTCAGGTCGTGTC-3′; down/5′-GCGCTTCCGGCCCAG-3′. To amplify *PRL-3* in xenograft samples, we use human-specific oligonucleotides to avoid the amplification of murine *PRL-3*: up/5′-GTGAAGGCCAAGTTCTGT-3′; down/5′-ACAAGGACTGGAGCCCGG-3′. For normalisation of *PRL-3* expression levels, we analysed the expression of human-specific *β*2-microglobulin, a housekeeping gene widely used in qPCR with CRC samples (Haller *et al*, 2004; Rubie *et al*, 2005; Abal *et al*, 2007; Menendez *et al*, 2008): up/5′-CCCACTGAAAAAGATGAG-3′; down/5′-CCTCCATGATGCTGCTT-3′. Human-specific E-cadherin was used as a control between xenografts and paired primary tumours to evaluate the influence of expression rates in epithelial cells because of the different phylogenetic origin of stromal cells (up/5′-CCTATTTTTCCCTCGACA-3′; down/5′-GCAATTCTGCTTGGATTC-3′). We used cyclophillin to check the stability of the *β*2-microglobulin expression in the entire group of samples (up/5′-TTCTTCGACATTGCCGT-3′; down/5′-TGCTGTCTTTGGGACC-3′). All of the experiments were performed in triplicate using two different retrotranscriptions.

### Immunohistochemical analysis

The 3 *μ*m slices of paraffin-embedded tissue were used. For antigen retrieval, the slides were boiled after deparaffinization in a pressure cooker for 2 min in citrated buffer (8.2 mM trisodium citrate and 1.98 mM citric acid, pH6), and endogen peroxidase was blocked with 3% H_2_O_2_ for 20 min. After blocking for 30 min with 1/5 dilution of goat serum, the primary antibodies were incubated for 1 h at room temperature. Primary antibodies were polyclonal antibodies for PRL-3 (rabbit anti-PRL-3, Zymed Laboratories, Invitrogen, Carlsbad, MA, USA, and rabbit anti-PRL-3, ab50276 Abcam, Cambridge, UK), diluted 1 : 500 in phosphate-buffered saline (PBS). Reaction was visualised using the EnVision antirabbit antibody system and developed using the DAB-Plus Kit (Dako, Copenhagen, Denmark). Slides were counterstained with Harry's modified haematoxylin. As a negative control, we used the EnVision antirabbit antibody system that displayed no reactivity against any antigen.

A PRL-3 related protein-blocking experiment was performed. Before the staining protocol, PRL-3 polyclonal antibodies were incubated for 1 h at room temperature with an excess of PRL-1 or PRL-2 or PRL-3 blocking protein (Abcam, Cambridge, UK). The antibody that is bound to the blocking protein is no longer available to bind to the epitope present in the protein on the tissue.

Blinded analysis of the slides was performed by two independent observers (NB and DGM).

### Western blot analysis

1 *μ*g of human recombinant PRL-1, PRL-2 and PRL-3 (Abcam, Cambridge, UK) was heated at 95°C for 5 min, electrophoresed through 18% SDS–polyacrilamide gel and transferred onto a nitrocellulose membrane. PRL-3 was immunodetected by using two different antibodies: rabbit anti-PRL-3 (Zymed Laboratories, Invitrogen, Carlsbad, MA, USA) and rabbit anti-PRL-3, ab50276 (Abcam, Cambridge, UK), both diluted 1 : 500 and incubated overnight at 4°C. Secondary antibody antirabbit IgG coupled with horseradish peroxidase was incubated for 30 min at room temperature. The ECL kit (Amersham General Electric) was used for detection.

### Statistical analysis

REST-XL^©^ relative expression software tool was used to evaluate PRL-3 expression in which expression ratios are tested for significance by a pair-wise fixed reallocation randomisation test (Pfaffl, 2001).

Moreover, independent samples *t*-test was applied in xenografts and mCRC samples, and paired samples *t*-test, in matched triplet samples and paired normal colonic mucosa/mCRC.

Association between PRL-3 expression and clinicopathological variables was assessed by the *χ*^2^-test. Survival was defined as the time from the date of primary tumour resection until death, being censored for patients who were alive at the time of the last follow-up. Survival curves were estimated using the Kaplan–Meier method. Cox's proportional hazard models were fitted to estimate hazard ratios (HRs), and 95% confidence interval (CI) and likelihood ratio tests were performed to assess the statistical significance of the variables.

Binary logistic regression was performed to identify the predictors for distant dissemination.

## Results

### PRL-3 is specifically expressed in tumour colorectal cells

**Figure 1:**
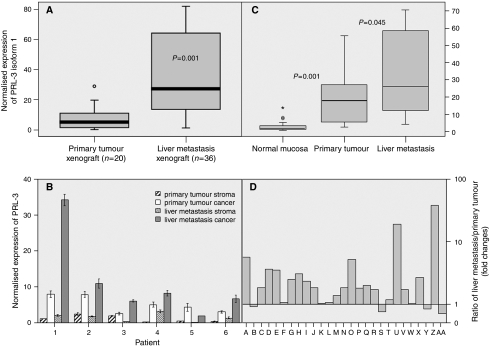
(**A**) Normalised expression of PRL-3 in xenografts determined by quantitative –PCR. Boxplots comparing PRL-3 mRNA expression in 20 human colorectal primary tumours and 36 liver metastases from CRC raised as xenografts in nu/nu Swiss mice. (**B**) Normalised expression of PRL-3 determined by Quantitative –PCR in paired samples of microdissected specimens. We analysed four different cell populations from six different patients: (i) primary tumour stromal cells, (ii) primary tumour cancer cells, (iii) hepatic metastases stromal cells and (iv) hepatic metastases cancer cells, obtained by means of Arcturus PixCell II Laser-Capture microdissection system. (**C**) Boxplots comparing PRL-3 mRNA normalised expression in 27 matched fresh-frozen samples of non-adjacent normal mucosa, primary tumour and liver metastases (22 synchronous and five metachronous). Comparing normal colonic mucosa and primary tumours, pair-wise fixed reallocation randomisation test (Pfaffl, 2001) showed significantly higher expression in carcinomas by the factor 23.133 (*P*<0.001). Besides, liver metastases displayed a higher expression than primary colorectal tumours (factor 1.983; *P*=0.045). (**D**) Bars displayed the overexpression of isoform 1 PRL-3 in 23 out of 27 liver metastases. Bars represent the ratio among PRL-3 normalised expression in liver metastases divided by PRL-3 normalised expression in its paired primary tumour. The ratio was expressed as fold changes. In all experiments, the level of PRL-3 expression was expressed as absolute expression normalising for *β*2-microglobuline as housekeeping control gene. All experiments were performed in triplicate using two different retrotranscriptions.

To investigate the specificity of PRL-3 expression in colorectal cancer cells, a set of xenografts from primary colorectal tumours (*n*=20) and LM from CRC (*n*=36) were analysed. Xenografts are characterised by the lack of human stroma providing a good model to study the specificity of the tumour cell population expression. Using human-specific oligonucleotides, PRL-3 expression was already detected in orthotopic xenografts derived from primary CRC, although at low levels (Figure 1A). Metastasis xenografts implanted in the mouse liver showed higher levels (7.2-fold; *U*-Mann–Whitney *P*<0.001)) when compared with primary CRC ones. Using oligonucleotides that selectively amplify the murine PRL-3, very low or absent PRL-3 expression was detected both in primary and LM xenografts (data not shown) disregarding a relevant contribution from the mouse stroma to the total amount of PRL-3 in the xenografts, even endothelial cells. To give further insight into the value of using xenografts for RNA expression purposes, we compared, as a control, the expression of E-cadherin between 20 xenografts derived from primary colorectal tumours and its paired human primary tumours. No differences were observed among the two groups (*P*=0.69; Supplementary Figure S1), ruling out a possible artefact of transplantation among species on the expression of cancer-involved proteins.

To further confirm that PRL-3 expression is specific of tumour cells, microdissection was performed in a set of six matched primary tumour/LM samples. Again, expression was observed in all primary tumour cancer cells and at a lower degree in the corresponding stroma. In five out of six cases, overexpression was evident in the metastasic lesion when compared with primary tumours (Figure 1B). Although some degree of expression was detected in the stroma, probably related to endothelial cells as reported earlier (Bardelli *et al*, 2003), expression levels were mainly residual.

### Both PRL-3 isoforms are overexpressed in the primaries. The overexpression is further selected during distal dissemination

To analyse whether PRL-3 overexpression was mainly a metastasis-specific event or was already present at significant levels in primaries, we analysed a set of 27 matched triplets of normal colonic mucosa/primary tumour/LM (22 synchronous, five metachronous). As depicted in Table 1 and figure 1C, PRL-3 isoform 1 was consistently overexpressed (23.1-fold) in primary tumours in relation to its paired normal mucosa (*P*=0.001). Isoform 2 was also upregulated in primary tumours, although at a lower intensity (three-fold when compared with paired normal mucosa; *P*=0.001), contributing to higher PRL-3 levels in primary tumours in relation to its counterpart normal mucosa.

Further upregulation of both PRL-3 isoforms was evidenced in matched LM (isoform 1, two-fold; *P*=0.045; isoform 2, 1.5-fold; *P*=0.003; Table 1). This overexpression was evident in 23 out of 27 LMs for isoform 1 (Figure 1D) and in 21 out of 27 LMs for isoform 2.

### PRL-3 expression levels in primary colorectal tumours associates with tumour aggressiveness

**Figure 2:**
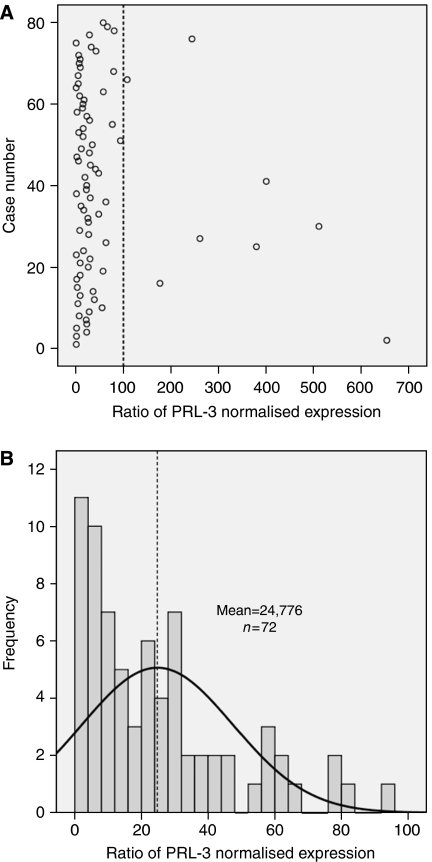
(**A**) PRL-3 normalised expression for 80 primary tumours adjusted for their counterpart normal mucoses. As values did not fit a normal distribution (Kolmogorov–Smirnov test *P*<0.0001), extremely high values plotted to the right of the dashed line were coded as ‘high expression’. Values on the left of the dashed line (72 patients) were evaluated to fit a normal distribution (Kolmogorov–Smirnov test *P*=0.113); therefore, in (**B**), we plotted values of PRL-3 expression for the normally distributed values. Dashed line designates the median value (cutoff) and separates two populations coded as ‘low expression’ and ‘high expression’. Then, 38 values were coded as high expression and 42 values as low expression.

**Figure 3:**
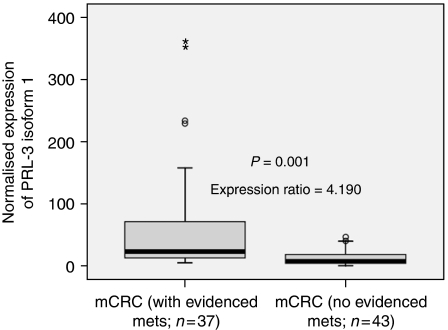
Normalised expression of PRL-3 in 43 metastatic colorectal carcinomas (29 stage IIIB and 14 stage IIIC) that did not develop distant metastasis for at least 60 months and 37 metastatic colorectal carcinomas (17 stage IIIB and 20 stage IIIC) that developed distant metastases determined by Quantitative PCR. Pair-wise fixed reallocation randomisation test showed a higher expression of PRL-3 in patients who developed liver or lung metastasis (*P*=0.001). The level of PRL-3 expression was expressed as absolute expression normalising for *β*2-microglobuline as housekeeping control gene.

We analysed a selected set of 80 stage III colorectal carcinomas with a similar proportion of recurrent (*N*=37) and non-recurrent (*N*=43) cases. To dichotomise a continuous variable, PRL-3 normalised expression for each of the 80 stage III primary tumours adjusted for their counterpart normal mucoses was calculated (Figure 2A). On account of the data that did not fit a normal distribution, outlier values were coded as ‘high expression’. Therefore, the cutoff was calculated with the values (*N*=72) that fit a normal distribution (Figure 2B). Accordingly, PRL-3 expression was coded as a categorical variable. Binary logistic regression showed that PRL-3 expression was a predictor of distant metachronous metastasis in univariate analysis (*P*=0.000; Table 2). In multivariate analysis, the high level of PRL-3 was the only independent predictor of metastasis (*P*<0.0001; OR: 9.791; Table 2). Consequently, 28 out of 38 primary tumours (73.7%) displaying high PRL-3 expression eventually developed liver or lung metastases, whereas 33 out of 42 patients (78.6%) showing low PRL-3 expression did not (*χ*^2^-test, *P*=0.000). When tumours were classified according to the presence or absence of metastases, the former showed a 4.190-fold average increase of PRL-3 expression (*P*=0.001; Figure 3). Finally, 24 out of 38 patients (63.15%) with high levels of PRL-3 expression displayed vascular or lymphatic invasion, with the difference being statistically significant in relation to low-expression cases (17 out of 42; *P*=0.035). These results point to a role of PRL-3 in the spreading of CRC metastasis when primary cancer cells intravasate into capillaries in agreement with Kato *et al* (2004).

**Figure 4:**
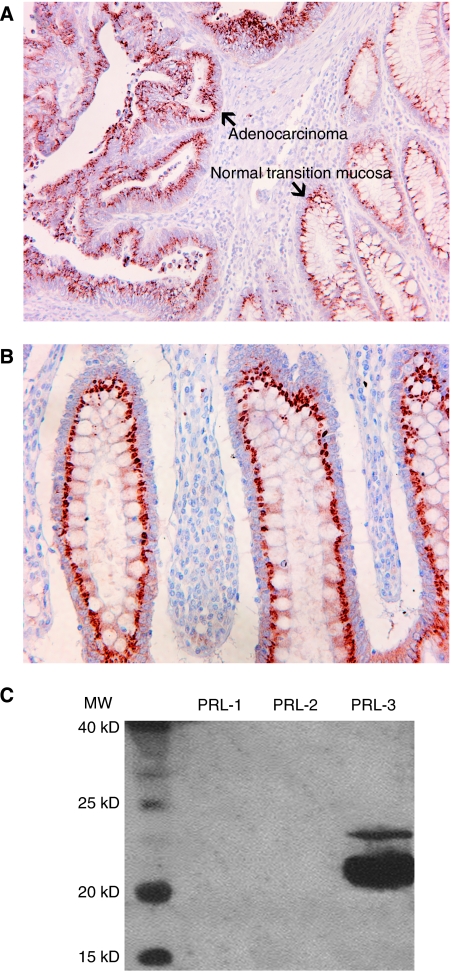
(**A**) Photomicrography ( × 200) showing staining with anti-PRL-3 antibody (1 : 500), both in the tumoral compartment and normal transition mucosa. (**B**) Photomicrography ( × 400) showing staining with anti-PRL-3 antibody (1 : 500) in a normal colonic mucosa. (**C**) Western blot analysis showing specificity of rabbit antihuman PRL-3 polyclonal antibody against PRL-3 in its denatured conformation (SDS–PAGE).

We also performed an immunohistochemical staining of PRL-3 in this set of stage III colorectal carcinomas. As shown in Figure 4, the PRL-3 antibody displayed an intense paranuclear and granular staining probably related to the Golgi apparatus or transport vesicles, both in the tumoral cells (Figure 4A) and normal colonic mucosa (Figure 4B). Unexpected staining of normal mucosa led us to check the specificity of this antibody performing IHC with a previous blocking assay with an excess of PRL-1 or PRL-2 or PRL-3. The staining persisted after blocking, and therefore the antibodies have no reactivity against PRL-1, PRL-2 and PRL-3 in their natural conformation, thus making the evaluation of PRL-3 overexpression with this immunohistochemical approach extremely difficult. On the other hand, Western blot showed no reactivity with PRL-1 and PRL-2 and strong staining for PRL-3 (Figure 4C).

### High levels of PRL-3 expression in colorectal primary tumours was associated with worse survival

**Figure 5:**
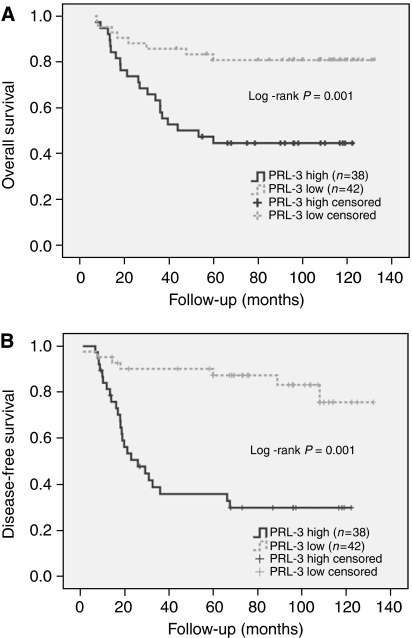
(**A**) OS (overall survival) and (**B**) DFS (disease-free survival) Kaplan–Meier survival plots according to PRL-3 expression status. Eighty primary metastatic colorectal tumours (46 stage IIIB and 34 stage IIIC) and their correspondent normal colonic mucosa were obtained from the Department of Pathology of the Hospital Universitari de Bellvitge. Among the 80 CRCs, we classify 43 CRC (29 stage IIIB and 14 stage IIIC) that did not develop distant metastases for at least 60 months and 37 CRCs (17 stage IIIB and 20 stage IIIC) that developed liver or/and lung metastases. All patients underwent surgical resection – 70 radical surgery and 10 palliative surgery. All patients received adjuvant chemotherapy treatment. The median follow-up was 107.30 months (range: 43–132). End points were local recurrence for DFS and cancer-related death or last follow-up for OS. At the end of the follow-up period 45out of 80 patients (56.25%) were alive without disease, six (7.5%) were alive with disease and 29 died of cancer. PRL-3 expression values were normalised for *β*2-microglobulin. Absolute expression of each tumour sample was adjusted for expression of its paired normal colonic mucosa. *P*-values correspond to the univariate model of Cox regression test and were considered significant if *P*⩽0.05.

As shown in Table 2 and Figure 5A, the expression of high levels of PRL-3 was associated in univariate analysis with poorer outcome (mean survival: 57.17±6.16 months for high expression *vs* 87.12±5.75 months for low expression; HR 3.639; CI: 1.607–8.24; *P*=0.002). Moreover, the expression of PRL-3 was also associated with a shorter DFS (mean DFS: 41.98±6.207 months for high expression *vs* 76.01±5.759 months for low expression; HR 5.926; CI: 2.54–13.805; *P*<0.0001; Figure 5B).

Besides, high PRL-3 expression was associated with poor overall survival. Among patients with low PRL-3 expression, 80.95% (four with disease) are alive, compared with only 44.738% (two with disease) in the high PRL-3 expression group. In multivariate analysis, the high level of PRL-3 was the only independent predictor of survival in our selected set of cases (*P*=0.006; HR: 3.322; CI: 1.405–7.852; Table 2) along with a stage for DFS (*P*<0.0001 for PRL-3 expression; *P*=0.020 for stage).

## Discussion

In this study, we show that phosphatase PRL-3 is an independent marker of aggressiveness and distant dissemination of locally advanced CRC and a useful tool to classify the accuracy of stage III CRC regarding the risk of distant relapse.

The first studies on PRL-3 expression in CRC reported that this phosphatase was restrictedly expressed in metastatic cancer cells but not in earlier stages of tumorigenesis (Bardelli *et al*, 2003), and stated that PRL-3 was undetectable in normal mucosa and non-metastatic CRC. Later, authors hypothesised that PRL-3 expression was required for the earlier phases of the metastatic process, such as those required for invasion into the lymphatics or vasculature (Kato *et al*, 2004). In addition, an immunohistochemical approach asserted that PRL-3 was a potential marker for LM of colorectal cancer and that it negatively influenced the prognosis of colorectal cancer patients (Peng *et al*, 2004), although it is questionable to involve PRL-3 in prognosis in non-consecutive series.

To date, there are no studies dealing with PRL-3 expression using the approach of human xenografts in nude mice. Xenografts are characterised by no contamination of human non-tumoral cells. Then, using oligonucleotides to amplify the specifically human sequence of the target gene (*PRL-3*) and housekeeping gene, we can ensure not only an enrichment of sample to almost 100% but also that specific expression is not masked for murine RNA. Such an approach leads us to determine an overexpression of PRL-3 in LMs seven-fold higher than in primary colorectal tumours, confirming previously reported descriptive results using other enrichment techniques (Saha *et al*, 2001). Giving strength to the xenograft approach, we evaluated the possible influence of tumour microenvironment on the expression rates of tumoral cells. Then, we compared, using human-specific oligonucleotides, E-cadherin expression between CRC xenografts and paired human primary tumours showing no differences, confirming the usefulness of xenografts to RNA expression purposes and, in particular, PRL-3 expression. Furthermore, we avoid the contribution of murine vasculature, although scarce, to PRL-3 levels reported by other authors (Bardelli *et al*, 2003).

The assessment of PRL-3 in microdissected matched samples confirmed that stromal cells (including carcinoma-associated fibroblast, inflammatory cells and endothelial cells essentially) scantly contributed to the total PRL-3 expression and suggested that the analysis of primary tumours and metastases samples without tumour cell enrichment could provide meaningful data about the role of PRL-3. This is especially relevant in the metastases analyses where stromal reaction is very abundant in the majority of cases.

Evaluation of PRL-3 in matched triplets normal mucosa, primary tumour and LMs displayed greater differences in expression in the step normal mucosa to primary tumour in comparison with the step primary tumour towards LM. Thus, most of the overexpression detected in metastases is already present in the corresponding primary tumours. Altogether, our findings further suggest that the phosphatase is required for the processes of migration and invasion both at the primary site and the secondary site where it is still selected. Finally, it must be emphasised that no differences were observed regarding synchronous and metachronous lesions, as suggested by other authors, although the limited sample size precludes drawing conclusions.

For the first time, we assessed the expression of PRL-3 isoform 2. PRL-3 exists in two isoforms – one of 173 amino-acid residues and the other of 148 amino acids. Isoform 2 is also upregulated in colorectal primary tumours approximately three-fold higher than in normal mucosa, but its function, as for isoform 1, remains to be elucidated.

Finally, we explored the hypothesis that PRL-3 could be used as a marker to select patients with locally advanced CRC (stages IIIB and IIIC) and predict those with higher risk to relapse in distant sites. In stage III colorectal carcinoma, approximately 70% of patients will develop distant metastases, mainly confined to the liver, during the course of 2 or 3 years after the resection of their primary tumour (Khatri *et al*, 2005). Our results point to a putative clinical value of the semiquantitative PRL-3mRNA level assessment as a prognostic marker in stage III tumours receiving adjuvant therapy. This technique is a good alternative to ISH and IHC and provides a more reliable quantitative assessment. Although Peng *et al* (2004) have shown that IHC can be a good alternative (, we have not been able to obtain reproducible immunostaining results using commercially available antibodies to date, as these antibodies also stained normal colonic mucosa as also reported by Peng *et al* (2004) using 3B6 monoclonal antibody. Although commercial antibodies are specific for PRL-3 in a denaturised linear conformation, as we observed by western blot, perhaps they cannot recognise the antigen in its native conformation, as immunostaining after blocking with the entire PRL-3 protein reproduces the same pattern.

In summary, the quantitative assessment of PRL-3 mRNA levels is an adequate tool, and better than IHC, to study routinely collected tumour samples, as overexpression is apparently restricted to the tumour cell compartment limiting the impact of the intensity of tumour stromal reaction on the final quantitative assessment. Moreover, PRL-3 overexpression is a common event in stage III colorectal primary tumours that is further selected during liver dissemination. PRL-3 expression correlates with tumour aggressiveness and is a promising predictor of distant metastases. Therefore, the assessment of PRL-3 expression will be of great interest to identify patients with higher risk of relapse who could benefit from a more exhaustive surveillance to anticipate the appearance of disseminated disease, or on the other hand, patients that have an unexpectedly good outcome despite the presence of locoregional metastases.

For the purpose of prognosis, it is mandatory to validate the cutoff value of PRL-3 expression in larger and consecutive series in the adjuvant setting.

## Figures and Tables

**Table 1:** Statistical analysis of PRL-3 expression (change fold) in matched triplet samples and stage IIIB-C primary tumours with evidenced metachronous metastasis (mCRC-met) and without evidenced metachronous metastasis (mCRC-nomet)

	**Expression ratio**	**Pair-wise randomisation test**	**Paired samples *t*-test**	**Independent samples *t*-test**
*Matched samples*				
Primary tumour/normal mucosa	23.133	0.001	<0.001	—
Liver metastasis/primary tumour	1.983	0.045	<0.001	—
				
*Stage IIIB-C primary tumours*				
mCRC-nomet/paired normal mucosa	7.743	0.001	<0.001	—
mCRC-met/paired normal mucosa	47.38	0.001	<0.001	—
mCRC-met/mCRC-nomet	4.190	0.001	—	0.004

REST-XL^©^ relative expression software tool was used to evaluate PRL-3 expression in which expression ratios are tested for significance by a pair-wise fixed reallocation randomisation test (Pfaffl, 2001), and plotted using standard error estimation through a complex Taylor algorithm. REST-XL^©^ performs 10 000 random reallocations of samples and controls between the groups, and counts the number of times the relative expression of the randomly assigned group is greater than the sample data.

Moreover, independent samples *T*-test was applied in mCRC samples, and paired samples *t*-test, in matched triplet samples and paired normal colonic mucosa/mCRC.

**Table 2:** Descriptive statistic, univariate and multivariate analyses for PRL-3 and clinicopathological variables

	**Descriptive**			**Univariate Cox's proportional hazard model**			**Univariate binary logistic regression**			**Multivariate Cox's proportional hazard model**			**Multivariate binary logistic regression**		
	***N***	**%**	**Mean survival**	**HR**	**CI**	***P*-value**	**OR**	**CI**	***P*-value**	**HR**	**CI**	***P*-value**	**OR**	**CI**	***P*-value**
*Gender*															
Female	40	50	78.76±6.8	1	0.672–2.948	0.366	1	0.683–4.022	0.263	1	0.54–2.46	0.714	1	0.483–4.072	0.533
Male	40	50	67.04±5.8	1.407			1.658			1.153			1.403		
															
*Tumour stage*															
IIIB	46	57.5	80.11±5.53	1	0.951–4.121	0.068	1	0.983–6.044	0.055	1	0.848–4.076	0.122	1	0.793–7.9	0.118
IIIC	34	42.5	63.14±7.26	1.98			2.437			1.859			2.503		
															
*Location*															
Colon	54	67.5	76.09±7.38	1	0.544–2.625	0.657	1	0.393–2.51	0.99	1	0.564–2.794	0.578	1	0.327–3.118	0.987
Rectum	26	32.5	71.36±5.68	1.195			1.006			1.255			1.009		
															
*Vascular or lymphatic invasion*															
No	39	48.8	82.63±6.01	1	0.866–3.886	0.113	1	0.928–5.607	0.072	1	0.484–2.544	0.805	1	0.392–3.792	0.732
Yes	41	51.3	63.64±6.41	1.834			2.282			1.110			1.219		
															
*PRL-3 expression*															
Low	42	52.5	87.12±5.75	1	1.607–8.24	0.002	1	3.65–28.81	<0.0001	1	1.405–7.852	0.006	1	3.308–28.98	<0.0001
High	38	47.5	57.17±6.16	3.639			10.267			3.322			9.791		

Cox's proportional hazard models were fitted to estimate hazard ratios (HRs), and 95% confidence interval (CI) and likelihood ratio tests were performed to assess the statistical significance of the variables.

Binary logistic regression was performed to identify predictors for distant dissemination.
