# Supplementary material for: PRL-3 is essentially overexpressed in primary colorectal tumours and associates with tumour aggressiveness
Source: Br J Cancer. 2008 Oct 28;99(10):1718–25. doi: 10.1038/sj.bjc.6604747 (PMC2584959; doi:10.1038/sj.bjc.6604747)

Supplementary figure S1

Human specific E-cadherin normalised expression of 20 human primary colorectal tumours raised as xenografts in nude mice and its paired human primary colorectal tumours. Pair wise Fixed Reallocation Randomisation Test showed no differences in E-cadherin expression between two groups (P=0.69). The level of PRL-3 expression was expressed as absolute expression normalising for β2-microglobuline as housekeeping control gene.

SUPPLEMENTARY FIGURE S1


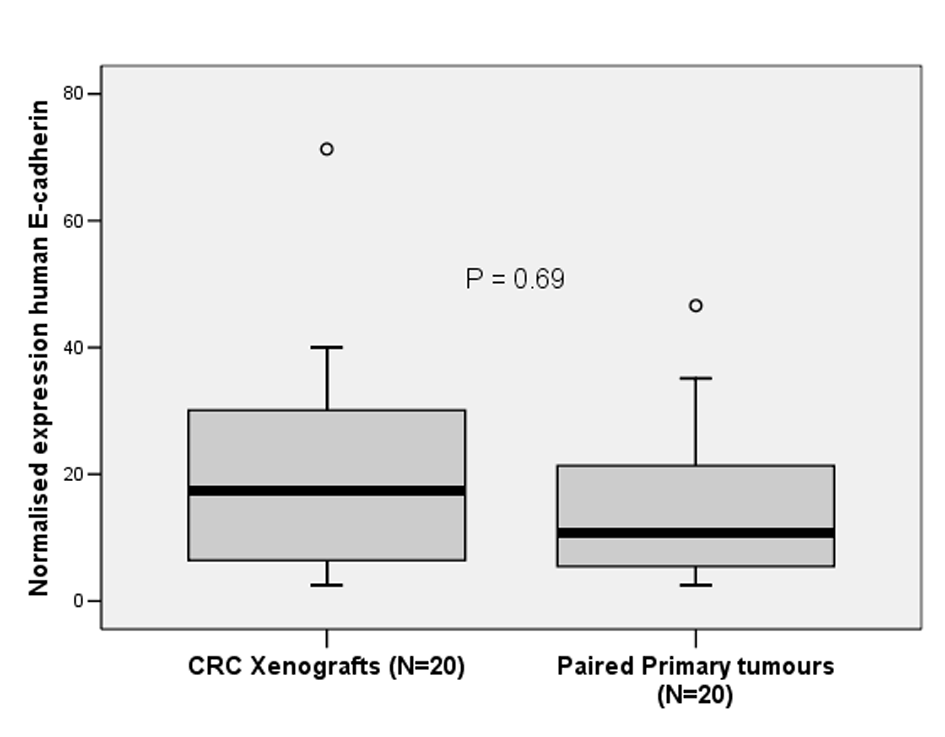

Supplement: Supplementary Figure S1 [file 6604747x1.doc]
